# Supplementary material for: Hadaean to Palaeoarchaean stagnant-lid tectonics revealed by zircon magnetism
Source: Nature. 2023 Jun 14;618(7965):531–6. doi: 10.1038/s41586-023-06024-5 (PMC10266976; doi:10.1038/s41586-023-06024-5)
Supplement: Supplementary file 1 — This file contains Supplementary Text and Supplementary References [file 41586_2023_6024_MOESM1_ESM.pdf]

nature portfolio

https://doi.org/10.1038/s41586-023-06024-5

Supplementary information

Hadaean to Palaeoarchaean stagnant-lid tectonics revealed by zircon magnetism

In the format provided by the authors and unedited

Hadean to Palaeoarchean stagnant lid tectonics revealed by zircon magnetism
Supplementary Information

Supplementary Table 1. SHRIMP U-Pb geochronology data

The Pb/Pb age results for each BGS zircon grain are described below.

BGS1-z3

Morphology and zoning: Euhedral prism with concentric, oscillatory zoning visible in cathodoluminescence (CL) images. Minor fractures. Age: Five analyses were carried out on four areas of zircon BGS1-z3 (one analysis was a down-hole replicate), targeting inner and outer zones. The weighted mean ^{207}Pb/^{206}Pb age of these five analyses is 3398 $\pm$ 21 Ma (95% confidence interval), mean square of weighted deviates (MSWD) = 2.3, probability of fit (PoF) = 0.06. This is interpreted as a reliable age for this grain.

BGS1-z4

Morphology and zoning: Euhedral prism with concentric, oscillatory zoning visible in CL images. Minor fractures. Age: Four analyses were carried out on separate areas of zircon BGS1-z4, targeting inner and outer zones. The weighted mean ^{207}Pb/^{206}Pb age of these analyses is 3396 $\pm$ 8 Ma (95% confidence interval), MSWD = 0.73, PoF = 0.53.

BGS1-z7

Morphology and zoning: Subrounded prism with broad sector zoning visible in CL images. Minor fractures. Age: Four analyses were carried out on separate areas of zircon BGS1-z7, targeting inner and outer zones. The weighted mean ^{207}Pb/^{206}Pb age of these analyses is 3383 $\pm$ 8 Ma (95% confidence interval), MSWD = 1.07, PoF = 0.36.

BGS1-z9

Morphology and zoning: Subrounded prism with broad sector zoning visible in CL images. The inner part of the grain is dark in CL (higher U) relative to a lighter 15-20 μm rim. See Fig. 2j-k. Age: Thirteen analyses were carried out on 11 areas of zircon BGS1-z9 (including down-hole replicates on one spot), targeting the core and the rim. The weighted mean ^{207}Pb/^{206}Pb age of six analyses of the core is 4091$\pm$18 Ma (95% confidence interval), MSWD = 2.2, PoF = 0.047. The weighted mean ^{207}Pb/^{206}Pb age of 4 analyses of the rim is 3881 $\pm$ 82 Ma (95% confidence interval), MSWD = 2.5, PoF = 0.06. This weighted mean consists of replicates on spot 11 and spot 3. Additional analyses of this rim (spots 2.2, 9, 10) were conducted but inclusion of these in the weighted mean result in excess scatter, suggesting disturbance to the isotopic system. The age of 3881 Ma should be considered the minimum age of a high temperature event that resulted in rim growth which we equate with the age of magnetization.

BGS1-11

Morphology and zoning: Euhedral prism with concentric, oscillatory zoning visible in CL images. Minor fractures. See Fig. 2g-h. Age: Eight analyses were carried out on separate areas of zircon BGS1-z11, targeting inner and outer zones. The weighted mean $^{207}Pb/^{206}Pb$ age of seven of these analyses is 3768 ± 9 Ma (95% confidence interval), MSWD = 1.2, PoF = 0.30. One analysis (111.7) was excluded as a statistical outlier.

BGS1-z12

Morphology and zoning: Subhedral to anhedral fragment with broad sector zoning in CL images. Minor fractures. Age: Four analyses were carried out on separate areas of zircon BGS1-12, targeting different sectors of the zonation. The weighted mean $^{207}Pb/^{206}Pb$ age of all analyses is 3404 ± 8 Ma (95% confidence interval), MSWD = 0.82, PoF = 0.48.

BGS1-z14

Morphology and zoning: Anhedral fragment with broad sector zoning in CL images. Minor fractures. See Extended Data Fig. 3b-c. Age: Four analyses were carried out on separate areas of zircon BGS1-z14, targeting different sectors of the zonation. The weighted mean $^{207}Pb/^{206}Pb$ age of all analyses is 3390 ± 25 Ma (95% confidence interval), MSWD = 1.5, PoF = 0.22.

BGS1-z15

Morphology and zoning: Subrounded prismatic zircon fragment. Oscillatory zoning in CL, fractured. Minor alteration along some zones. Age: Four analyses were carried out on separate areas of zircon BGS1-z15. The weighted mean $^{207}Pb/^{206}Pb$ age of all analyses is 3385 ± 27 Ma (95% confidence interval), MSWD = 3.6, PoF = 0.013. This low probability of fit suggests that this grain may have a disturbed U-Pb isotopic system. Alternatively one of these analyses may just be a statistical outlier. We exclude the youngest analysis to calculate a weighted mean $^{207}Pb/^{206}Pb$ age of the remaining three of 3393 ± 35 Ma (95% conf., MSWD = 2.3, PoF = 0.10) which is equated with the age of magnetization.

BGS2-z1 Morphology and zoning: Subrounded prism with fine-scale oscillatory zoning visible in CL images. Age: Four analyses were carried out on separate areas. The weighted mean $^{207}Pb/^{206}Pb$ age of these analyses is 3422 ± 11 Ma (95% confidence interval), MSWD = 0.42, PoF = 0.74.

BGS2-z2

Morphology and zoning: Euhedral prism with fine-scale oscillatory zoning in CL images. Grain has a 10-20 μm wide rim characterized by broader zoning. Core of grain exhibits minor alteration along some zones. Age: Four analyses of the finely zoned zircon core give a weighted mean $^{207}Pb/^{206}Pb$ age of 4011±12 Ma (95% confidence interval, MSWD = 0.43, PoF = 0.73), however two analyses of the broadly zoned rim give a weighted mean $^{207}Pb/^{206}Pb$ age of 3621 ± 18 Ma (95% confidence

interval, MSWD = 0.73, PoF = 0.39). One older analysis targeting the rim was excluded from this calculation as it is interpreted to represent a mixed age with the core. The age of the rim is considered the best estimate of the time of a high temperature event and thus magnetization.

BGS2-z3

Morphology and zoning: Euhedral prism with concentric, oscillatory zoning visible in cathodoluminescence (CL) images. Fractured, with minor alteration visible along some zones. Age: Three analyses were carried out on unfractured areas of the grain. The weighted mean ^{207}Pb/^{206}Pb age of these three analyses is 3467 ± 7 Ma (95% confidence interval, MSWD = 0.44, PoF = 0.64). Despite the fracturing, there is no evidence of isotopic resetting.

BGS2-z4

Morphology and zoning: Euhedral prism with faint, broad, concentric zoning visible in cathodoluminescence (CL) images. Minor fractures. Age: Four analyses were carried out across the grain. The weighted mean ^{207}Pb/^{206}Pb age of these four analyses is 3388 ± 20 Ma (95% confidence interval, MSWD = 1.5, PoF = 0.21).

BGS1-z5

Morphology and zoning: Euhedral prism with broad, straight zoning visible in cathodoluminescence (CL) images. Minor fractures. Age: Four analyses were carried out across a variety of wide zones in this grain. The weighted mean ^{207}Pb/^{206}Pb age of these four analyses is 3393 ± 9 Ma (95% confidence interval, MSWD = 0.39, PoF = 0.76).

BGS2-z7

Morphology and zoning: Euhedral prism concentric zoning visible in cathodoluminescence (CL) images. Minor fractures and alteration/inclusions in the very center of the grain. Age: Four analyses were carried out across the grain. The weighted mean ^{207}Pb/^{206}Pb age of these four analyses is 3470 ± 10 Ma (95% confidence interval, MSWD = 0.90, PoF = 0.44).

BGS2-z8

Morphology and zoning: Equant grain, possibly the tip of a prismatic zircon grain with oscillatory zoning visible in CL. Minor fractures. Age: Four analyses were carried out across the grain. The weighted mean ^{207}Pb/^{206}Pb age of these four analyses is 3488 ± 10 Ma (95% confidence interval, MSWD = 0.53, PoF = 0.66).

BGS2-10

Morphology and zoning: Prismatic grain with sector zoned core and distinct concentrically zoned rim (up to 15 μm wide). Minor fractures cut both core and rim. See Extended Data Fig. 3k-l. Age: Four analyses were conducted on the core. The weighted mean ^{207}Pb/^{206}Pb age of three of them is 4075 ± 86 Ma (95% confidence interval, MSWD = 2.3 PoF = 0.10). One analysis (spot

210.6) is excluded from this calculation as it is relatively young (ca. 3977 Ma) and discordant (8%). Only only two analyses were possible for the rim. These yielded non-reproducible $^{207}Pb/^{206}Pb$ ages of 3226 ± 26 Ma (2σ confidence interval) and 3262 ± 26 Ma (2σ confidence interval). The age of the BGS is bracketed between 3298± 6 Ma (2σ) for the unit below and 3279 ± 8 Ma (2σ) for the unit above[1−2]. We interpret the growth of the rim, which we equate with the age of magnetization, to be related to ca. 3.3 Ga magmatism recorded by tuffs in the sedimentary sequence which subsequently experienced Pb-loss after 3.22 Ga (resulting in 3.26 Ga and 3.22 Ga spot results).

BGS2-z11
Morphology and zoning: Euhedral prism with concentric zoning. Minor fractures. Age: Three analyses were carried out across the grain. The weighted mean $^{207}Pb/^{206}Pb$ age of these analyses is 3552 ± 8 Ma (95% confidence interval, MSWD = 0.41, PoF = 0.66).

BGS2-z13
Morphology and zoning: Equant, euhedral prism with concentric zoning. Minor fractures. Age: Three analyses were carried out across the grain. The weighted mean $^{207}Pb/^{206}Pb$ age of these analyses is 3384 ± 12 Ma (95% confidence interval, MSWD = 0.63, PoF = 0.53).

BGS2-z14
Morphology and zoning: Equant fragment with patchy/convolute zoning. Minor fractures. See Fig. 2d-e. Age: Four analyses were carried out across the grain. The weighted mean $^{207}Pb/^{206}Pb$ age of these analyses is 3890 ± 10 Ma (95% confidence interval, MSWD = 0.23, PoF = 0.88).

BGS2-z15
Morphology and zoning: Euhedral prism with concentric zoning. Fractured. Alteration is present along the margin of the grain. Age: Three analyses were carried out across the grain, avoiding fractures and alteration. The weighted mean $^{207}Pb/^{206}Pb$ age of these analyses is 3760 ± 33 Ma (95% confidence interval, MSWD = 1.6, PoF = 0.21).

BGS2-z17
Morphology and zoning: Euhedral prism with concentric zoning. Bright CL inner region and dark CL outer zone however all zones are concordant with one another (not cross-cutting). See Extended Data Fig. 3e-f. Age: Four analyses were carried out across the grain, including the apparent bright CL "core" and dark CL "rim". The weighted mean $^{207}Pb/^{206}Pb$ age of these analyses is 3372 ± 10 Ma (95% confidence interval, MSWD = 1.13, PoF = 0.34). There is no statistically distinguishable difference in the ages of these zones and thus this is interpreted as a reliable age for this grain.

BGS2-z19
Morphology and zoning: Equant fragment with sector zoning. Age: Four analyses were carried out across the grain, targeting different sectors. The weighted mean $^{207}Pb/^{206}Pb$ age of these analyses

is 3374 ± 10 Ma (95% confidence interval, MSWD = 0.7, PoF = 0.55).

BGS2-21

Morphology and zoning: Tip of oscillatory zoned, prismatic zircon. Strongly fractured and altered along some zones. Age: Given the degree of fracturing, only 1 analysis spot was possible on this grain. It returned a ^{207}Pb/^{206}Pb age of 3480 ± 18 Ma (2σ), which we interpret as a reliable record of the age of the grain.

BGS2-z22

Morphology and zoning: Fragment of prismatic zircon with oscillatory zoning. Minor fractures. Age: Four analyses were carried out across the grain. The weighted mean ^{207}Pb/^{206}Pb age of these analyses is 3394 ± 21 Ma (95% confidence interval, MSWD = 2.0, PoF = 0.11).

BGS5-z1

Morphology and zoning: Subrounded prismatic zircon with broad concentric zoning. Minor fractures. Age: Four analyses were carried out across the grain. The weighted mean ^{207}Pb/^{206}Pb age of these analyses is 3554 ± 6 Ma (95% confidence interval, MSWD = 1.0, PoF = 0.39).

BGS5-z5

Morphology and zoning: Subrounded prismatic zircon with convolute zoning. Fractured with minor alteration along fracture surfaces. Age: Four analyses were carried out across the grain. The weighted mean ^{207}Pb/^{206}Pb age of these analyses is 3398 ± 17 Ma (95% confidence interval, MSWD = 1.8, PoF = 0.14).

BGS5-z6

Morphology and zoning: Euhedral, prismatic zircon with oscillatory zoning. Fractured. Age: Three analyses were carried out across the grain, avoiding fractures. The weighted mean ^{207}Pb/^{206}Pb age of these analyses is 3516 ± 10 Ma (95% confidence interval, MSWD = 0.85, PoF = 0.43).

BGS5-z7

Morphology and zoning: Euhedral, prismatic zircon with oscillatory zoning. Fractured. Age: Three analyses were carried out across the grain, avoiding fractures. The weighted mean ^{207}Pb/^{206}Pb age of these analyses is 3299 ± 9 Ma (95% confidence interval, MSWD = 0.34, PoF = 0.71).

BGS5-z8

Morphology and zoning: Euhedral, prismatic zircon with oscillatory zoning. Fractured, with minor alteration near tip of grain. Age: Five analyses were carried out across the grain, avoiding fractures. The ^{207}Pb/^{206}Pb ages from these five analyses range from 3314 Ma to 3401 Ma. The younger analyses do not correspond with the outer parts of the grain which would have suggested the presence of an older core and younger rim. Instead the non-reproducibility of age indicates a disturbances

to the isotope systematics of this grain, which we infer occurred at relatively low temperature. The best estimate for the crystallization age of this zircon is that the oldest analysis (3401 ± 22 Ma, 2σ) which we equate with the magnetization age.

BGS5-z12

Morphology and zoning: Euhedral, prismatic zircon with concentric and convolute zoning. Fractured, with minor alteration (tip of grain). Age: Seven analyses were carried out across the grain, targeting the range of zoning styles and avoiding fractures. The $^{207}Pb/^{206}Pb$ ages from these five analyses range from 3782 Ma to 3609 Ma, the youngest of which is highly discordant (46%). We infer that the younger ages are the result of Pb-loss at relatively low temperature. The best estimate for the crystallization age of this zircon is that the weighted mean $^{207}Pb/^{206}Pb$ age of the oldest 4 analyses at 3748 ± 25 Ma (95% confidence interval, MSWD = 2.6, PoF = 0.053, and we equate this with the age of magnetization.

BGS5-z14

Morphology and zoning: Euhedral, prismatic zircon with oscillatory zoning. Fractured. Age: Three analyses were carried out across the grain, avoiding fractures. The weighted mean $^{207}Pb/^{206}Pb$ age of three of these analyses is 3488 ± 44 Ma (95% confidence interval, MSWD = 3.7, PoF = 0.02). The fractures throughout this grain prohibited placing more than 3 analytical spots, so while the probability of fit falls outside our 5% threshold, we consider this a reliable age for this grain given its small number of analyses.

BGS5-z15

Morphology and zoning: Equant euhedral zircon with oscillatory zoning. The truncation of some zoning gives the appearance of a core and a rim to this grain. Minor fractures. Age: Four analyses were carried out across the grain, two from the "core" and two from the rim. The weighted mean $^{207}Pb/^{206}Pb$ age of three of these analyses is 3484 ± 44 Ma (95% confidence interval, MSWD = 1.4, PoF = 0.24). It excludes one slightly older analysis from the "rim" which is interpreted as an analytical outlier. Given the good statistical fit of this data, the observed zoning does not represent a core/rim relationship with distinctly different ages. The weighted mean age is therefore interpreted as a reliable age for this grain.

BGS5-z17

Morphology and zoning: Euhedral, prismatic zircon with oscillatory zoning. Minor fractures. See Extended Data Fig. 3h-i. Age: Four analyses were carried out across the grain, avoiding fractures. The weighted mean $^{207}Pb/^{206}Pb$ age of these analyses is 3487 ± 10 Ma (95% confidence interval, MSWD = 0.71, PoF = 0.54).

BGS5-z18

Morphology and zoning: Subhedral fragment with convolute zoning and a CL dark (relatively higher U) outer zone. Minor fractures. Age: Four analyses were carried out across the grain, including on the higher U outer zone. The weighted mean $^{207}Pb/^{206}Pb$ age of all four analyses is 3392 ± 15 Ma (95% confidence interval, MSWD = 0.38, PoF = 0.77). The high probability of fit indicates that there is no statistical difference in the ages of the inner and outer zones, thus is interpreted as a reliable age for this grain.

BGS5-z19

Morphology and zoning: Equant, euhedral prism with concentric zoning. Minor fractures. Age: Four analyses were carried out across the grain. The weighted mean $^{207}Pb/^{206}Pb$ age of all four analyses is 3389 ± 17 Ma (95% confidence interval, MSWD = 1.3, PoF = 0.27).

Supplementary Information References

1. Byerly, G. R., Kröner, A. Lowe, D. R., Todt, W. & Walsh, M. W., Prolonged magmatism and time constraints for sediment deposition in the early Archean Barberton greenstone belt: evidence from the Upper Onverwacht and Fig Tree groups. *Precamb. Research* **78**, 125-138 (1996).
2. Decker, N. B., Byerly, G. R., M. Thompson Stiegler, Lowe, D. R. & Stefurak, E. High resolution tephra and U/Pb chronology of the 3.33–3.26Ga Mendon Formation, Barberton Greenstone Belt, South Africa. *Precamb. Res.*, **261**, 54-74 (2015).
